# Prognostic utility of basaloid differentiation in oropharyngeal cancer

**DOI:** 10.1186/1916-0216-42-57

**Published:** 2013-12-19

**Authors:** Timothy Cooper, Vincent Biron, Ben Adam, Alexander C Klimowicz, Lakshmi Puttagunta, Hadi Seikaly

**Affiliations:** 1 Division of Otolaryngology - Head and Neck Surgery, Department of Surgery, University of Alberta, 1E4 University of Alberta Hospital, Edmonton, AB T6G 2B7, Canada; 2 Department of Laboratory Medicine & Pathology, University of Alberta, 5B2 University of Alberta Hospital, Edmonton, AB T6G 2B7, Canada; 3 Department of Oncology, University of Calgary, Tom Baker Cancer Centre, 1331-29 Street NW, Calgary, AB T2N 4 N2, Canada

**Keywords:** Basaloid differentiation, HPV, p16, Hematoxylin, Eosin, Oropharynx, Squamous cell carcinoma, Outcomes, Survival

## Abstract

**Background:**

Human papillomavirus (HPV) is recognized as the key risk factor for a distinct subset of oropharyngeal squamous cell carcinoma. P16 is a reliable, sensitive surrogate marker for HPV and confers a positive prognostic advantage. Basaloid differentiation on hematoxylin and eosin (H&E) staining is anecdotally noted by some pathologists to be associated with p16 positivity. This association, however, has not been adequately quantified in the literature, nor has the prognostic implications of basaloid differentiation been described.

**Objectives:**

1) To correlate the H&E staining feature of basaloid differentiation with p16 positivity in oropharyngeal cancer. 2) To investigate the prognostic utility of basaloid differentiation in oropharyngeal cancer survival.

**Methods:**

Retrospective cross-sectional study of all patients diagnosed with and treated for oropharyngeal cancer at a single tertiary cancer center from 2002 to 2009. Tissue microarrays (TMAs) were generated from 208 oropharyngeal tumor specimens stained with H&E and immunohistochemical markers. These oropharyngeal TMAs were utilized in several previous publications. Samples were scored for basaloid differentiation by a pathologist blinded to the p16 result. A multivariate survival analysis with Cox-regression and Kaplan-Meier survival analysis was performed.

**Results:**

In the 208 samples, basaloid differentiation correlated with p16 positivity (Spearman’s rho 0.435). Basaloid differentiation and p16 positivity were both independent predictors of improved survival. The 5 year disease specific survival (DSS) was 73% for p16 positive tumors and 35% for p16 negative tumors (p < 0.001). Similarly, the 5 year DSS of basaloid differentiated tumors was 74% compared to 41% for non-basaloid tumors (p = 0.001). Patients with p16 positive and basaloid differentiated tumors had the best survival outcomes with a 5 year DSS of 80%.

**Conclusions:**

Basaloid differentiation is a feature on H&E which correlates with p16 positivity and is a simple, inexpensive, independent, positive prognostic indicator of comparable magnitude to p16 status. Due to the added prognostic value of basaloid differentiation, this feature should be routinely reported by qualified pathologists.

## Background

Human papillomavirus (HPV) is recognized as the key risk factor for a distinct subset of oropharyngeal squamous cell carcinoma [1-4]. The proportion of oropharyngeal cancer attributable to HPV is increasing dramatically and is now thought to account for approximately 70% of oropharyngeal squamous cell carcinoma [1,5-7].

There are numerous assays for the detection of HPV in tumor cells. These include immunohistochemistry (IHC) for p16 protein, polymerase chain reaction (PCR) and in-situ hybridization techniques for detection of viral DNA, and reverse transcriptase PCR (RT-PCR) for viral mRNA [8,9]. The gold standard for HPV detection is RT-PCR for viral E6 and E7 mRNA, although it is not routinely performed [9]. Commonly, p16 IHC is performed [10]. P16 is a cyclin-dependent kinase inhibitor which is overexpressed in cells infected with HPV [11,12]. Studies have shown that p16 IHC is a reliable, sensitive surrogate marker for HPV and confers a positive prognostic advantage [6,8,10,13].

Hematoxylin and eosin (H&E) staining is routinely performed on all biopsy and surgical specimens submitted for pathology. It is an inexpensive stain with readily available results. The classical description of HPV-related oropharyngeal cancer histology is non-keratinizing and basaloid differentiated [3,5,9,13,14]. Keratinization is the feature that has been focused on in the literature to date and in pathology reports. Basaloid differentiation is anecdotally noted by some pathologists to be associated with p16 positivity. This association, however, has not been adequately quantified in the literature [3,11,12,15], nor has the prognostic implications of basaloid differentiation been described.

The purpose of this study was twofold:

1) to quantify the association of the H&E marker of basaloid differentiation with p16 IHC in oropharyngeal squamous cell carcinoma.

2) to investigate the prognostic utility of the H&E marker of basaloid differentiation in oropharyngeal squamous cell carcinoma.

## Methods

This is a retrospective cross-sectional study set in a regional head and neck cancer treatment center. Approval was obtained from the University of Alberta Health Research Ethics Board prior to the commencement of the study. Patients were identified through the Alberta Cancer Registry in a prospective manner from 2002 to 2009 for inclusion in the study. Patient demographics, staging, treatment, and survival data were collected.

All patients diagnosed and treated with oropharyngeal squamous cell carcinoma in Edmonton, Alberta between 2002 and 2009 were eligible for inclusion. Each patient required a core or tissue biopsy to be performed for use in a tissue microarray (TMA). Included patients were treated with curative intent with any combination of cancer treatment modalities including surgery, chemotherapy, and radiation.

Patients and their associated TMAs were excluded if their cancer was treated with palliative intent or inadequate tissue was obtained for assessment of H&E staining features or determination of p16 status.

### TMA construction

TMAs were constructed with formalin-fixed paraffin-embedded (FFPE) tumor tissue from either pre-treatment biopsies or primary surgery. A pathologist reviewed the blocks and excluded cases with inadequate tissue for future diagnosis. FFPE blocks were marked by a pathologist for TMA construction. The TMAs were constructed with duplicate or triplicate cores of FFPE blocks as per the TMA protocol described by Klimowicz et al. [16]. These TMAs had been utilized in previous studies conducted by the authors.

### Immunohistochemistry

IHC for p16 was performed using the diaminobenzidine (DAB) staining method as previously reported by Lau et al. [10]. In accordance with previously established standards in the literature, p16 positivity was defined as high intensity staining in greater than 70% of cells scored manually by a pathologist.

### Histologic analysis

H&E features of each TMA, including basaloid differentiation and keratinization, were scored by a pathology resident and confirmed by a staff head and neck pathologist. Both individuals interpreting the H&E staining features were blinded to the p16 status of the TMAs. Basaloid differentiation was defined as the presence of two of three features associated with basaloid differentiation that were identifiable on TMA including peripheral palisading, high nuclear to cytoplasmic ratio, and solid growth pattern.

### Statistical analysis

Spearman’s correlation was used to calculate the correlation between p16 status and basaloid differentiation. Multivariate analysis was performed using Cox proportional hazards regression for the variables basaloid differentiation, p16 status, age, gender, and treatment modality. Kaplan-Meier survival analysis was performed to calculate disease specific survival (DSS) within subgroups based on histologic features and IHC staining. Comparison of proportions was performed using χ^2^ or Fisher’s exact test as appropriate and continuous data using Student’s *t*-test. Statistical significance was accepted as P <0.05 in all cases.

Statistical analysis was performed using SPSS Version 21 (IBM). The database was initially constructed using Excel 2010 (Microsoft) and converted to SPSS in order for data analysis to be performed.

## Results

A total of 208 patients and their TMAs were included in the study. The mean age was 58.4 years (range 32–95 years). There was a male predominance with 161 men and 47 women. 189 patients presented with advanced stage disease (stage 3 or 4) compared to only 19 with early stage disease (stage 1 and 2). Nodal disease was present in 172 patients on presentation. Surgery followed by chemotherapy and radiation was the most common treatment. Of the 208 tumor specimens, 111 (53%) were p16 positive and 97 (47%) were p16 negative. Eighty-four (40%) demonstrated basaloid differentiation while 124 (60%) were non-basaloid. A breakdown of demographic information as well as staging, tumor characteristics, and treatment modalities used is presented in Table 1.

**Table 1:** Clinicopathologic characteristics, staging, and treatment of patients based on p16 and basaloid differentiation stratification

**Characteristic**	**All**	**B**	**NB**	**P**	**+P16**	**-P16**	**P**
**n**	208	84	124	-	111	97	-
**Mean age**	58.4	57.4	59.1	0.28	55.3	62.0	<0.01
**Gender (male)**	161	64	97	0.73	88	73	0.49
**P16 Status**							
Positive	111	69	42	<0.01	-	-	-
Negative	97	15	82		-	-	
**Staging (%)**							
Advanced	91	94	89	0.19	97	84	<0.01
Early	9	6	11		3	16	
**T Staging (%)**							
T1	19	23	17	<0.01	21	17	0.10
T2	27	36	21		28	26	
T3	31	33	30		35	27	
T4	23	9	33		16	31	
**N Stage (%)**							
N0	14	7	18	0.04	6	24	<0.01
N positive	86	93	82		94	76	
**Treatment (%)**							
Primary surgery	75	77	73	0.54	72	68	0.06
Primary RT	25	23	27		28	32	
Surgery + CRT	37	52	27	<0.01	51	21	<0.01
Surgery + RT	23	14	28		19	27	
CRT	17	17	18		14	21	
RT	8	6	10		5	11	
Surgery	15	11	18		10	21	

n = number of patients. Primary RT = radiation therapy +/− Chemotherapy. Primary surgery = Surgery +/− radiation therapy +/− Chemotherapy. B = basaloid differentiated. NB = non-basaloid. CRT = chemoradiation. RT = radiation. P values calculated using χ^2^or Fisher’s exact test for categorical data and two tailed t-test for continuous data.

Subgroup analyses comparing patients with or without basaloid differentiation and p16 positivity are also shown (Table 1). Patients who were p16 positive were younger than those who were p16 negative with a mean age of 55.3 ± 10.1 years compared to 62.0 ± 11.2 years (p < 0.01). There were no significant differences in gender distribution between groups. Patients who had p16 positive tumors were more likely to present with advanced stage disease (p < 0.01) and have positive nodal status (p < 0.01). Basaloid differentiated tumors were more likely to be p16 positive than non-basaloid tumors (p < 0.01). Non-basaloid differentiated tumors had more advanced T staging (p < 0.01) while basaloid differentiated tumors were more likely to have positive nodes (p = 0.04), with no significant difference in advanced compared to early stage disease. There were statistically significant differences between groups with regards to the treatment modalities used (p < 0.01) with p16 positive and basaloid differentiated patients being more frequently treated with surgery followed by chemoradiation.

Basaloid differentiation correlated with p16 positivity using Spearman’s correlation (Table 2), with a correlation coefficient of 0.435 (p < 0.001). Multivariate survival analysis with Cox proportional hazards regression gave statistically significant values for p16 positivity and basaloid differentiation as independent predictors of survival (Table 3). The calculated hazard ratios were 0.455 for basaloid differentiation, and 1.356 for p16 negativity. Treatment with either chemoradiation or radiation alone were statistically significant predictors of mortality compared to surgery followed by chemoradiation, which was used as a reference (p < 0.0001).

**Table 2:** Spearman’s correlation of basaloid differentiation with p16 positivity

**Characteristic**	**Spearman’s rho correlation coefficient**	**P**
Basaloid differentiation	0.435	<0.001

**Table 3:** Multivariate survival analysis with Cox proportional hazards regression

**Variable**	**HR**	**95% CI**	**P**
Basaloid differentiation	0.46	0.27-0.76	0.003
p16 negative	1.36	1.04-1.77	0.025
**Patient variables**			
Male gender	0.78	0.45-1.33	0.36
Age	1.02	1.00-1.04	0.5
**Treatment (Surgery + Chemoradiation reference)**			
Surgery + RT	1.31	0.63-2.73	0.461
Chemoradiation	3.51	1.76-6.99	<0.0001
Radiation	8.46	4.07-15.80	<0.0001

HR = hazard ratio. CI = confidence interval.

**Figure 1:**
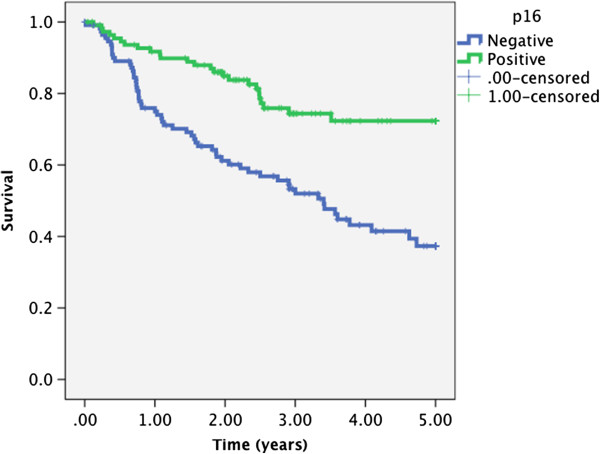
Kaplan-Meier analysis of disease specific survival in 208 patients according to p16 status (p < 0.001, log rank test).

**Figure 2:**
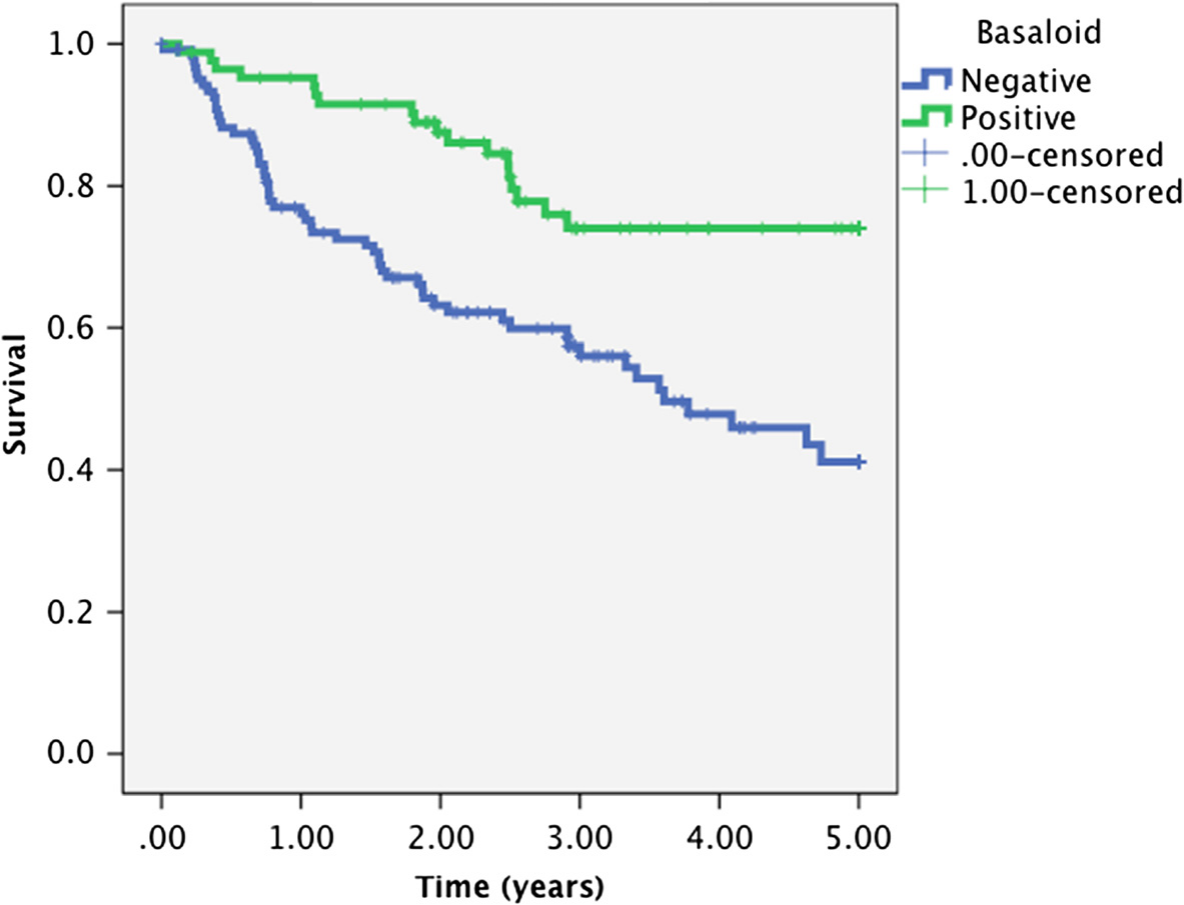
Kaplan-Meier analysis of disease specific survival in 208 patients according to basaloid differentiation (p = 0.001, log rank test).

Five year DSS calculated using Kaplan-Meier analysis for p16 positive tumors was 73% compared to 35% in p16 negative tumors (p < 0.001) (Figure 1). Similarly, 5 year DSS was 74% in basaloid differentiated patients compared to 41% in patients with non-basaloid tumors (p = 0.001) (Figure 2).

**Figure 3:**
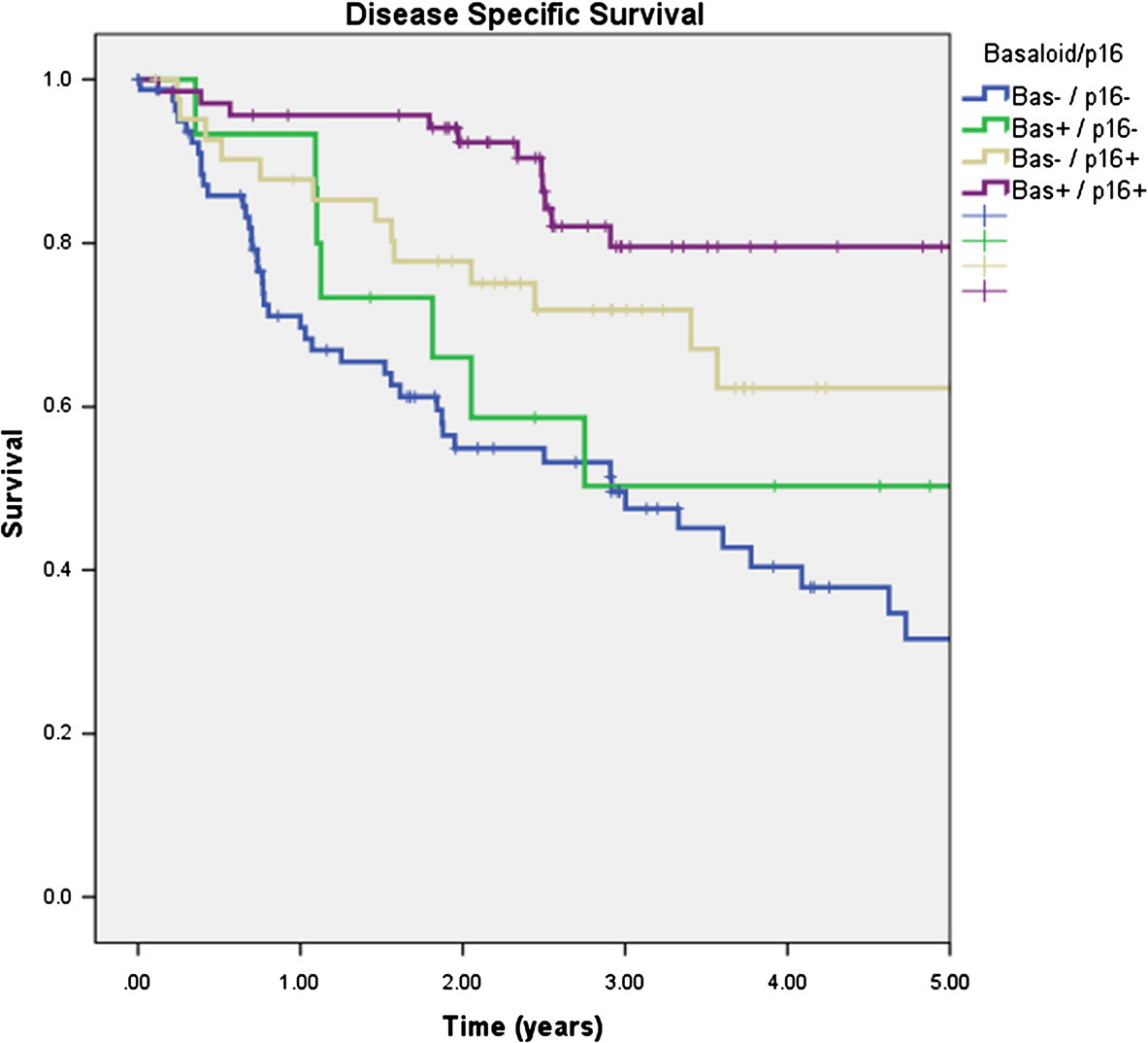
**Kaplan-Meier analysis of disease specific survival in 208 patients according to basaloid differentiation and p16 status (p < 0.001, log rank test).** Bas- = non-basaloid. Bas + =basaloid differentiated.

By combining basaloid differentiation and p16 status in Kaplan-Meier analysis, DSS could be further stratified (Figure 3). Those patients with basaloid differentiated, p16 positive tumors had a 5 year DSS of 80%, compared to 62% in non-basaloid, p16 positive patients, 50% in basaloid differentiated, p16 negative patients, and 32% in non-basaloid, p16 negative patients (p < 0.001).

## Discussion

Patients in our series who had p16 positive tumors were predominantly male, younger, and had more advanced staged disease with positive nodes; these findings are similar to what others have reported in the literature [1,17]. Patients with basaloid differentiated tumors did not have more advanced staging but were more likely to have nodal disease as well. Basaloid differentiation is a feature that has been identified as being associated with HPV-related oropharyngeal cancer [3,5,9,11,13,14]. In this study, basaloid differentiation was strongly correlated with p16 positivity but could not consistently predict p16 positivity; as such, it could not replace p16 as a surrogate marker for HPV positivity.

Basaloid differentiation is associated with HPV in oropharyngeal cancer and has been looked at in small numbers by several authors. Laco et al. [15] found that 17 of 21 HPV positive oropharyngeal cancer specimens were also basaloid differentiated. Mendelsohn et al. [11] found that 8 of 17 p16 positive and only 4 of 11 HPV positive by in situ hybridization head and neck cancers exhibited basaloid differentiation. Similarly, Gillison et al. [3] found that HPV positive oropharyngeal cancer was more likely to be basaloid differentiated. Basaloid differentiation was found in 11 of 34 HPV positive oropharyngeal tumors. However, about one third of tumor specimens did not have data available on basaloid differentiation in this study. Also, Hafkamp et al. [12] reported that 4 of 9 HPV positive oropharyngeal cancer specimens showed basaloid differentiation. Previously reported evidence of an association between basaloid differentiation and p16 positivity in oropharyngeal cancer consists primarily of small studies, some of which were not specific to the oropharynx. Our series is the largest to date to quantify basaloid differentiation in oropharyngeal cancer and its relation to p16 status.

We found basaloid differentiation to be a significant positive prognostic indicator in oropharyngeal squamous cell carcinoma. Patients with basaloid differentiated tumors had a 33% greater 5 year DSS compared to non-basaloid tumors. This finding has not previously been shown in a large series of patients. The positive prognostic implications of p16 have been well documented [10,15,18,19]. The magnitude of survival advantage with basaloid differentiation was comparable to that of p16 positivity in this study. Patients who were p16 positive had a 38% greater 5 year DSS compared to p16 negative patients. Based on these results, comparable survival prognostication could be obtained from either basaloid differentiation or p16 IHC. This has immediate practical applications to regions of the world that do not have access to p16 IHC or other HPV-related assays.

Further prognostic information is provided by combining basaloid differentiation with p16 status. Patients with basaloid differentiated, p16 positive tumors had the best survival outcomes, followed by non-basaloid and p16 positive tumors, then basaloid and p16 negative tumors, and lastly non-basaloid and p16 negative tumors. Due to the added prognostic value of basaloid differentiation, we are of the opinion that this feature should be routinely reported by qualified pathologists.

There were some limitations in this study. The retrospective nature of the study means that not all factors such as Eastern Cooperative Oncology Group (ECOG) Performance Status [20] and Charlson Comorbidity Index (CCI) [21] were taken into account. In addition, smoking status and alcohol consumption data were unreliable and thus not included in our multivariate analysis. There is, therefore, potential for bias within the groups, which may impact outcomes. In addition, the treatment modalities used were not equally distributed between groups and this could be another confounding factor beyond our control. One possible explanation for this is that basaloid differentiated and p16 positive patients were more likely to have nodal disease and may have been treated more aggressively with surgery followed by chemoradiation for this reason. All patients were identified from and treated at a single institution and although our institution is responsible for treatment of all oropharyngeal cancer within the region, our outcomes and treatment modalities may not be representative of other centers. Some patients were not able to be included in the study due to inadequate tissue availability for analysis. We were unable to perform real-time PCR analysis for HPV which would provide additional HPV status information. This would involve RNA isolation from the tissue samples and cDNA amplification which was not possible with the FFPE specimens used in this study.

## Conclusions

Risk stratification and prognostication based on tumor molecular characteristics is rapidly expanding in head and neck cancer. The focus has been on adding additional significant molecular markers to the array in order to gain further prognostic information and guide management. There is additional value and information to be obtained from a process that is already routinely performed, H&E staining. Basaloid differentiation in oropharyngeal cancer is an easy and inexpensive predictor of survival. The significant survival advantage based on basaloid differentiation alone is a novel finding for which the molecular basis should be further explored.

## Abbreviations

CCI: Charlson Comorbidity Index; DSS: Disease specific survival; ECOG: Eastern Cooperative Oncology Group; FFPE: Formalin-fixed paraffin-embedded; HPV: Human papillomavirus; H&E: Hematoxylin and eosin; IHC: Immunohistochemistry; PCR: Polymerase chain reaction; RT-PCR: Reverse transcriptase polymerase chain reaction; TMA: Tissue microarray.

## Competing interests

The authors have no financial or non-financial competing interests to disclose.

## Authors’ contributions

TC contributed with data collection, data analysis, writing and revision of the manuscript. VB contributed with conception and design, data collection, data analysis, and critical revision of the manuscript. BA contributed with data collection and data analysis. AK contributed with TMA construction and optimization of p16 staining and quantification. LP contributed with conception and design, data collection, and critical revision of the manuscript. HS contributed with conception and design, data collection, data analysis, writing and revision of the manuscript. All authors have given final approval of the version to be published.
